# Vaccination timeliness and associated factors among preterm infants at a tertiary hospital in Uganda

**DOI:** 10.1371/journal.pone.0221902

**Published:** 2019-09-06

**Authors:** Irene Nakatudde, Joseph Rujumba, Flavia Namiiro, Ali Sam, Jamir Mugalu, Philippa Musoke

**Affiliations:** 1 Department of Paediatrics and Child Health, College of Health Sciences, Makerere University, Kampala, Uganda; 2 Mulago National Referral Hospital, Kampala, Uganda; 3 Clinical Epidemiology Unit, College of Health Sciences, Makerere University, Kampala, Uganda; 4 Makerere University-John Hopkins University Research Collaboration, Kampala, Uganda; University of Campania, ITALY

## Abstract

**Background:**

Preterm infants are at increased risk of infections including vaccine preventable diseases. Therefore, timely vaccination is crucial to ensure adequate disease protection. Information on whether preterm infants are vaccinated according to chronological age as recommended is limited in low-income countries.

**Objectives:**

We evaluated the timeliness of vaccination and associated factors among preterm infants at Mulago hospital, Uganda.

**Methods:**

We conducted a mixed methods study between July 2016 and April 2017. Vaccination dates of preterm infants aged 6–24 months were obtained from child health cards. Additional data were collected using a questionnaire. Five key informant interviews with health workers and two focus group discussions with caregivers were conducted. Cox regression analysis was used to identify factors associated with vaccination timeliness. Qualitative data was transcribed and analysed manually using content thematic approach.

**Results:**

We enrolled 350 preterm infants, with a median age of 8.4 months (IQR 6.8–10.8). Less than half, 149/350 (42.6%) of infants received all vaccines within the recommended time range. Timely vaccination was highest for BCG (92%) and lowest for OPV (45.4%). Untimely vaccination was highest for vaccines administered at 6 weeks (DPT 1, PCV 1 and OPV 1) compared to other vaccines in the EPI schedule. Delivering from home or private clinics and vaccine stock-out were significantly associated with untimely BCG and OPV 0 vaccination. Low maternal education level and being very preterm were associated with untimely DPT 1 and DPT 3 receipt. Admission and long stay in the neonatal unit were associated with untimely DPT 1 receipt while extreme low birth weight was associated with untimely DPT 3 vaccination. Increasing parity was associated with untimely measles vaccination. Qualitative findings revealed that lack of knowledge and poor attitudes of health workers and caregivers, gaps in documentation of vaccination status and inadequate communication by health workers hindered timely vaccination.

**Conclusion:**

More than half of preterm infants attending a specialised clinic at Mulago National Referral hospital in Uganda did not receive vaccines within the recommended time range. Specific strategies to improve vaccination timeliness in preterm infants are needed especially among the extremely low birth weight, very preterm and those with prolonged hospitalisation.

## Introduction

Vaccination is one of the most cost-effective interventions in promoting child survival worldwide, averting two to three million child deaths from vaccine preventable diseases (VPDs) annually [1]. However, despite the high global vaccination coverage of 85% some children especially in developing countries still experience delays in receiving their routine vaccines, which increases their risk of acquiring VPDs [2]. Timely vaccination is even more important for preterm infants due to an immature immune system that increases their susceptibility to infections. Worldwide, nearly 13 million babies are born prematurely annually, with a gestational age of less than 37 completed weeks. Approximately 60% of preterm births occur in South East Asia and sub-Saharan Africa [3, 4] In Uganda, approximately 200,000 preterm births occur every year [3, 5]. Given, the increased risk of infection among preterm infants, they require early and adequate protection from diseases. The role of maternal immunisation during pregnancy in reducing the burden of VPDs such as tetanus, influenza and pertussis among new-borns cannot be overly emphasized [6–9]. However, maternal immunisation alone may not offer adequate protection against VPDs in preterm infants, given that the largest proportion of maternal antibodies (IgG) are transferred during the last trimester [8]. As such, preterm infants who already have an immature immune system may experience VPDs with increased frequency and severity during infancy if vaccines are not administered on time [10, 11]. For example, reported cases of pneumococcal diseases, pertussis and pertussis related hospitalisations and complications in developed countries are more common and severe in low-birth weight and preterm infants [10, 12, 13]. Moreover, passively acquired maternal antibodies wane to undetectable levels as early as 4 months of life [8]. Therefore, timely and complete receipt of vaccines among preterm infants is required to increase the window of protection against VPDs especially in the first year of life. Currently, the World Health Organisation (WHO) recommends that preterm infants be vaccinated according to chronological age as other infants without correction for gestational age or birth weight. The only exception to this is hepatitis B vaccination in infants weighing less than 2,000g because of a documented reduced immune response [14, 15]. Despite evidence that vaccines are safe and produce and adequate immune response in preterm infants [10, 11, 15], several studies in developed countries have shown that preterm infants are immunised with significant delay and at times vaccinations may be incomplete especially for those with lower birth weights (<2,500g) [13, 16–19]. The delay in vaccinating preterm infants could be a reflection of vaccine hesitancy. According to WHO, vaccine hesitancy defined as “the reluctance or refusal to vaccinate despite the availability of vaccines” is among the ten threats to global health in 2019 [20, 21]. The reasons why people choose not to vaccinate their children remain complex, but some factors have been documented in both developed and developing countries [20, 22–25]. A systematic review of vaccine hesitancy in low and middle-income countries documented concern about fear of harmful events following immunisation and distrust of vaccination programs as the most common factors influencing vaccination behaviour [22]. In addition, studies have revealed that vaccine hesitancy significantly increases the odds of untimely vaccination [23, 24, 26].

Uganda introduced the Expanded Program on Immunisation (EPI) in 1983, with the mandate to ensure that every child is fully vaccinated with high quality vaccines against tuberculosis, polio, diphtheria, pertussis, tetanus and measles [27]. Since then, other vaccines including hepatitis B vaccine (2002), *Haemophilus influenza type b* (Hib) (2002), pneumococcal conjugate vaccine (PCV) (2013), inactivated polio vaccine (IPV) (2016) and more recently rotavirus vaccine (2018) have also been introduced. Preventive child health services such as routine immunisation are available in both private and public health sectors in Uganda [28]. The majority of people in the country access these services through the public health sector [28]. Public health services are provided through the basic National Minimum Health Care Package (UNMHCP) at all levels of Uganda’s decentralised health system [28, 29]. Immunisation services are available free of charge at all health centres (HC) IIS, IIIs, IVs (health sub-district), general hospitals, regional referral hospitals and the national referral hospitals with the exception of HC 1 (Village Health Teams) [28]. Despite a steady improvement in Uganda’s vaccination coverage from 64% (2006) to 85% (2017) [30], outbreaks of VPDs such as measles remain rampant [31], which in part could be a reflection of incomplete and untimely vaccination. Current vaccination coverage with the third dose of DPT3 in Uganda is estimated at 85% [30]. However, information on vaccination coverage specific to preterm infants is not available, given that immunisation data is aggregated for both term and preterm infants. Furthermore, although several studies regarding the vaccination of preterm infants have been conducted in developed countries [16, 18, 32], studies in developing countries are limited. To our knowledge, no published information is available with regard to vaccination of preterm infants in Uganda. Therefore, this study evaluated the timeliness of vaccination and associated factors among preterm infants at Mulago hospital, Uganda.

## Methods

### Study setting

The study was conducted at the Mulago hospital preterm clinic from July 2016 to April 2017. Mulago hospital is one of the two National Referral hospitals in Uganda and is a teaching hospital for Makerere University, College of Health Sciences. The hospital provides specialised follow-up care for preterm infants after discharge from the special care baby unit (SCBU), a level III neonatal unit of the hospital through the outpatient preterm clinic. On average 1,500 babies that are discharged from the SCBU, attend the preterm clinic annually and approximately 30 infants attend the clinic daily. The clinic is held twice a week, on Mondays and Thursdays and the babies are reviewed every1-3 months until two years of age. One paediatrician, a nurse and a paediatric resident doctor run the clinic. During follow up visits, nutrition status, growth and development, vaccination status, and neurodevelopment are assessed and caregivers are guided on the care of the preterm infant. During the study period, the EPI was providing the following vaccines; BCG (Bacille de Calmette-Guérin), four doses of oral polio vaccine (OPV), three doses of the pentavalent vaccine protecting against diphtheria, pertussis, tetanus, hepatitis B and *Haemophilus influenzae* type b (Hib) disease and measles vaccine. Immunisation at Mulago hospital is only carried out on weekdays. All preterm infants admitted to the SCBU receive BCG and OPV 0 vaccines at birth if clinically stable. At discharge, the date of administration of the given vaccine is recorded on the discharge letter. Prior to discharge, caregivers are advised by the midwife to take the preterm infant to a health facility near home for missed and/or subsequent vaccines. At discharge, preterm infants are not issued with child health cards; instead caregivers are advised to get these cards from health facilities where they will receive the subsequent vaccines. Caregivers are also given an appointment date to return for follow up in the preterm clinic of the hospital.

### Study design

This was a cross-sectional study with both quantitative and qualitative methods of data collection and utilising a sequential explanatory design. The quantitative study was conducted first from 11^th^ July 2016 to 23^rd^ March 2017 followed by the qualitative study during April 2017.

### Quantitative study

#### Study population

Preterm infants aged 6–24 months attending the preterm clinic of Mulago hospital with a child health card (immunisation card) and whose parent(s) consented.

#### Sample size

Sample size was estimated using Kish Leslie formula for cross-sectional studies, based on the proportion of low birth weight infants who had delayed vaccination in Lima, Peru (65%) [17]. The calculated sample size was 350, with a precision of 0.05, 80% power and 95% confidence interval.

#### Study procedure and data collection

The study team consisted of the principal investigator and two nurses (research assistants). The research assistants approached all parents attending the preterm clinic with their infants on clinic days. They provided information about the study to the parents or guardians. The research assistants then identified infants eligible for the study and obtained written informed consent from their parents or guardians. Preterm infants aged 6–24 months were eligible for inclusion in the study. Full term infants, preterm infants whose parents had not come with an immunisation card at the time of the clinic visit or did not consent to participate in the study were excluded. Participants were enrolled consecutively until the desired sample size was attained.

The information obtained from the child health card and hospital discharge letter included; date of birth, gestational age at birth, birth weight, place of birth and vaccination dates for the different vaccine antigens. During the face-to-face interview with the parent or guardian the research assistants used a pre-tested structured questionnaire to obtain detailed information on social, economic, demographic and household characteristics of the preterm infant. These included; maternal age, antenatal care (ANC) attendance, parental education level, parental employment status, religion, ethnicity and length of stay in the SCBU. All interviews were conducted on the day of the clinic visit and lasted about 20–30 minutes.

#### Outcome variable

The outcome “timeliness” was defined as the time to receipt of a vaccine, calculated in days by subtracting the date of birth from date of vaccination of the infant. Vaccinations were timely (on time) if they were received with in the World Health Organisation recommended time ranges (Table 1) [33, 34].

**Table 1:** The Ugandan expanded programme on immunisation schedule (2016).

Recommended age	Vaccinations	WHO recommendation
Birth	BCG, OPV 0	BCG: birth–8 weeks OPV 0: birth–4 weeks
6 weeks	DPT-HepB-Hib 1 PCV 1, OPV 1	4 weeks– 2 months
10 weeks	DPT-HepB-Hib 2 PCV 2, OPV 2	8 weeks– 4 months
14 weeks	DPT-HepB-Hib 3 PCV 3, OPV 3	12 weeks– 6 months
9 months	Measles	38 weeks –12 months

#### Data management

Completed data forms were checked for completeness and accuracy by the principal investigator daily and kept in a secure locker. Data was double entered in Epidata v. 3.1 (Centers for Disease Control and Prevention, Atlanta, GA) and validated to check for any inconsistencies. All errors were corrected and the cleaned data was exported to STATA 13 (STATA Corporation, College Station, Texas, USA) for analysis.

### Data analysis

**Descriptive analysis.** The maternal, paternal and preterm infant characteristics were summarised using descriptive statistics such as frequencies and proportions for categorical variables. Continuous variables were summarised using means (standard deviations) or medians (interquartile range), depending on the distribution of the data. We categorised the outcome into two levels, i.e. infants *that received vaccines on time* (received vaccine within recommended ranges) and those that *did not receive vaccines on time* (received the vaccine so early, late or missed), to appreciate the proportion of infants that did not receive their vaccines with in the recommended time ranges.

**Analytical analysis.** To determine the factors associated with vaccination timeliness, the outcome “timeliness” was defined as the time to receipt of each vaccine was used. Therefore, Cox regression analysis model was used. The assumptions of Cox proportional hazard models were tested using Scale Schoenfeld residual plots. Scaled Schoenfeld residual test and the extended model fitted with time varying covariates. There was no time dependence of the Hazard Ratio. The assumptions were fulfilled using all the above tests. We used Cox proportional hazard model at bivariate analysis. Following a stepwise approach, all independent variables that had a p-value less than 0.2 at bivariate analysis were considered for multivariate analysis. Variables already known to be associated with vaccination timeliness were also considered for multivariable analysis irrespective of the p-value obtained at bivariate analysis. At multivariable analysis, all variables with the p-value of ≤ 0.05 were considered statistically significant. No interaction amongst variables or confounding was observed. The final model was tested for goodness of fit using a graph of Nelson Aalen cumulative hazard versus Cox-Snell residual. This showed an exponential relationship indicating that the model was a good fit. This analysis was done for BCG, OPV 0, DPT 1, DPT 3 and measles vaccine.

### Qualitative study

#### Focus group discussions

Eighteen purposively selected caregivers of preterm infants with delayed vaccination participated in two focus group discussions (FGDs) at the hospital. One group consisted of 8 participants aged 15–24 years and the other had 10 participants that were ≥ 25 years. A research assistant experienced in qualitative research moderated the discussions using a focus group guide with prompts while the principal investigator took notes. The discussions focused on participants’ understanding of a preterm infant, when a preterm infant should be immunised and the reasons why some preterm infants were not immunised on time. All FGDs were audio recorded and lasted approximately 60–90 minutes. Discussions were conducted in the local language (Luganda) and were transcribed in English by the qualitative researcher.

#### Key informant interviews

Five key informant interviews (KIIs) with health workers caring for preterm infants for more than 6 months at the hospital were conducted. These included, one paediatrician, two nurses and two paediatric resident doctors. An interview guide with probes was used to explore the delivery of immunisation services at Mulago hospital and perceived barriers to timely vaccination of preterm infants. Two research assistants conducted the interviews in English; one moderated the discussion while the other took notes. All interviews were audio recorded and transcribed by the research assistant experienced in conducting qualitative research. Each interview lasted 30–45 minutes.

#### Data management and analysis

Data was analysed manually using content thematic approach [35] by two experienced qualitative researchers. Organising themes and sub-themes were derived from the data. The main themes related to delayed vaccination were; preterm, parent and health system related factors. Direct quotations from participants were used to present the study findings.

#### Ethics considerations

The study was approved by the Makerere University School of Medicine Research and Ethics Committee (SOMREC), REC REF 2016–064. Written informed consent was obtained from all parents or guardians of preterm infants and from all health workers who participated in the key informant interviews in this study.

## Results

### Quantitative results

#### Socio-demographic characteristics of preterm infants and their parents

**Fig 1:**
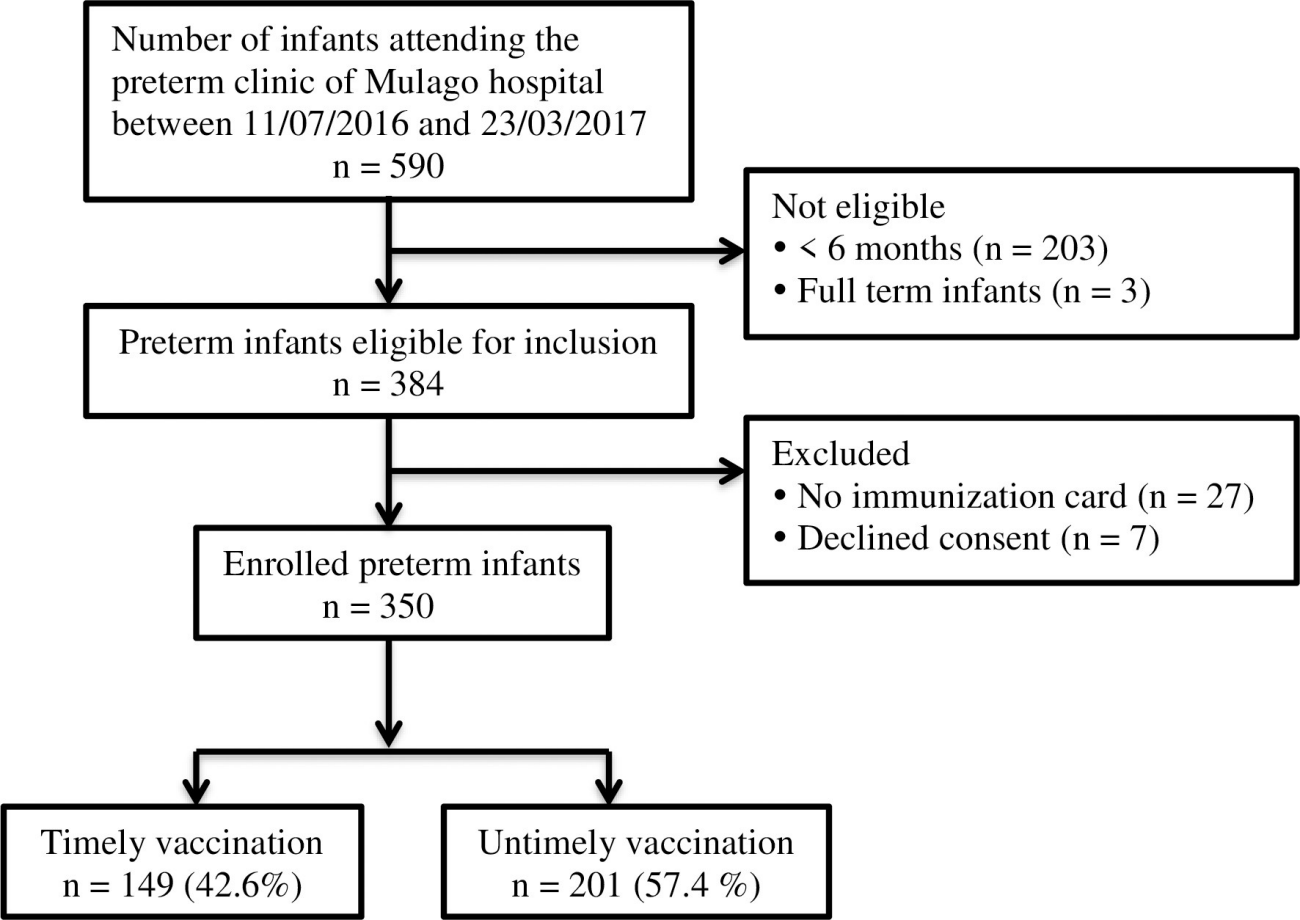
Flow diagram of the study population with inclusion and exclusion criteria.

Five hundred and ninety infants attending the preterm clinic were screened, and 384 were eligible for inclusion in the study. We enrolled 350 infants; the response rate was (91.1%) (Fig 1).

The median age of the preterm infants was 8.4 months (IQR 6.8–10.8) and 183/350 (52.3%) were female. About half of the infants were very preterm (28–32 weeks) 178 (50.9%), 145 (41.4%), were late preterm (32–37 weeks) and 27 (7.7%) were extremely preterm (< 28 weeks) (Table 2). The median age of the mothers was 27 years (IQR 23–32) with 11 (3%) below 18 years of age. The majority of mothers 341/350 (97.4%) had attended at least one antenatal care visit and delivered in hospital 307/350 (87.7%) (Table 2).

**Table 2:** Characteristics of preterm infants aged 6–24 months and their parents at Mulago Hospital, Kampala, Uganda (N = 350).

Variable	Frequency (n)	Percentage (%)
**Gender**		
Female	183	52.3
**Gestation age at birth**		
Late preterm (32–37 weeks)	145	41.4
Very preterm (28–32 weeks)	178	50.9
Extremely preterm (< 28 weeks)	27	7.7
**Birth weight**		
Normal (≥ 2.5 kg)	5	1.4
LBW (< 2.5 kg)	178	50.9
Very LBW (< 1.5 kg)	154	44
Extremely LBW (< 1kg)	13	3.7
**Delivery place**		
Hospital	307	87.7
Home or clinic	43	12.3
**Length of SCBU stay**		
≤7 days	187	53.4
>7 days	163	46.6
**Admission to SCBU**		
Yes	346	98.9
**Re-admission to SCBU**		
Yes	68	19.4
**Multiple births**		
Yes	95	27.1
**Maternal age**		
≤18 years	11	3.2
19–24 years	111	31.7
≥ 25 years	228	65.1
**Parity**		
≤ 2 births	207	59.1
≥ 3 births	143	40.9
**Attended ANC**		
Yes	341	97.4
**Maternal education level**		
Primary and none	77	22
Secondary	218	62.3
Tertiary and higher	55	15.7
**Maternal employment status**		
No	205	58.6
**Experienced vaccine stock out**		
Yes	128	36.6

#### Timeliness of vaccination of preterm infants

**Timeliness of vaccination excluding measles vaccine**

Overall, less than half 149/350 (42.6%) of all preterm infants received all vaccines with in the recommended time ranges. The proportion of preterm infants with timely vaccination was different for each vaccine (Table 3). For individual vaccines, BCG was the timeliest vaccine, with 92% of infants receiving it on time, while OPV was the least timely received vaccine (45.4%). Overall, 201/350 (57.4%) preterm infants had untimely vaccination (did not receive at least one vaccine within the recommended time range) (Table 3). Of these, 176/350 (50.3%) received at least one vaccine late and 87/350 (24.9%) missed at least one vaccine. One, 1/350 (0.3%) infant received their vaccines early, these included DPT 1, PCV 1, OPV 1, DPT 3 and PCV 3 (Table 4).

**Table 3:** Timeliness of vaccination among 350 preterm infants aged 6–24 months at Mulago Hospital, Kampala, Uganda.

Vaccine	Timeliness	Frequency (n)	Percentage (%)
**Overall**	On time	149	42.6
	Not on time	201	57.4
**BCG**	On time	322	92
	Not on time	28	8
**OPV**	On time	159	45.4
	Not on time	191	54.6
**DPT-HepB-Hib**	On time	202	57.7
	Not on time	148	42.3
**PCV**	On time	186	53.1
	Not on time	164	46.9
Measles*	On time	112	89.6
	Not on time	13	10.4

Measles* N = 125 (Preterm infants ≥ 9 months)

**Table 4:** Timeliness of each vaccine antigen among 350 preterm infants aged 6–24 months at Mulago Hospital, Kampala, Uganda.

Vaccination	Vaccine	Timely	Untimely			
Schedule		Vaccination	Vaccination			
		Total	Total	Late	Missed	Early
		n (%)	n (%)	n (%)	n (%)	n (%)
**Birth**	**BCG**	322 (92)	28 (8)	27 (7.7)	1 (0.3)	0 (0.0)
**Birth**	**OPV 0**	284 (81.1)	66 (18.9)	23 (6.6)	43 (12.3)	0 (0.0)
**6 weeks**	**OPV 1**	210 (60.0)	140 (40.0)	139 (39.7)	0 (0.0)	1 (0.3)
**10 weeks**	**OPV 2**	280 (80.0)	70 (20.0)	59 (16.9)	11 (3.1)	0 (0.0)
**14 weeks**	**OPV 3**	268 (76.6)	82 (23.4)	33 (9.4)	49 (14.0)	0 (0.0)
**6 weeks**	**DPT 1**	221(63.1)	129 (36.9)	128 (36.6)	0 (0.0)	1 (0.3)
**10 weeks**	**DPT 2**	288 (82.3)	62 (17.7)	56 (16.0)	6 (1.7)	0 (0.0)
**14 weeks**	**DPT 3**	290 (82.9)	60 (17.1)	33 (9.4)	26 (7.4)	1 (0.3)
**6 weeks**	**PCV 1**	208 (59.4)	142 (40.6)	137 (39.1)	4 (1.2)	1 (0.3)
**10 weeks**	**PCV 2**	276 (78.9)	74 (21.1)	63 (18.0)	11 (3.1)	0 (0.0)
**14 weeks**	**PCV 3**	275 (78.6)	75 (21.4)	33 (9.4)	41 (11.7)	1 (0.3)
**9 months**	Measles*	112 (89.6)	13 (10.4)	3 (2.4)	4 (3.2)	6 (4.8)

Measles* N = 125 (Preterm infants ≥ 9 months)

The proportion of preterm infants with untimely vaccination was highest for the vaccines that are administered at 6 weeks; PCV 1 142/350 (40.6%), OPV 1 140/350 (40.0%) and DPT 1 129/350 (36.9%) compared to other vaccines in the EPI schedule (Table 4). Among the untimely vaccinations, most were received late, compared to those that were not received at all (missed) and those that were received early (Table 4). More preterm infants were late for the vaccines administered at 6 weeks OPV 1 139/350 (39.7%), PCV 1 137/350 (39.1%) and DPT 1 128/350 (36.6%) compared to those given at 10 and 14 weeks. On the other hand, more preterm infants did not receive (missed) the vaccines administered at 14 weeks OPV 3 (14%), PCV 3 (11.7%) and DPT 3 (7.4%) compared to those given at 6 and 10 weeks (Table 4).

#### Timeliness of measles vaccine administration

One hundred and twenty five preterm infants were eligible for measles vaccine. Of these, 112 (89.6%) received the vaccine with in the recommended time range (Tables 3 & 4). Thirteen infants had untimely measles vaccination, of these, 6 received the vaccine early, 4 were not vaccinated and 3 received the vaccine late (Table 4).

#### Factors associated with untimely vaccination of preterm infants

S1 Table shows the characteristics that were associated with untimely vaccination at bivariate analysis for the individual vaccines.

At multivariate analysis, delivering from home or a private clinic and experiencing vaccine stock out were significantly associated with untimely BCG and OPV 0 receipt. Being very preterm and a low maternal education level were associated with untimely DPT 1 and DPT 3 vaccination. Admission and long stay in the SCBU were associated with untimely DPT 1 receipt while extreme low birth weight was associated with untimely DPT 3 vaccination. Increasing parity was significantly associated with untimely measles vaccination (Table 5).

**Table 5:** Factors associated with untimely vaccination of preterm infants aged 6–24 months at Mulago Hospital, Kampala, Uganda.

Variable	Unadjusted HR	P-value	Adjusted HR	P-value
	(95% CI)		(95% CI)	
	**BCG and OPV0**			
**Delivery place**				
Hospital	1		1	
Home or clinic	0.72 (0.52–0.99)	0.047	0.71 (0.51–0.98)	0.042
**Vaccine stock out**				
No	1		1	
Yes	0.66 (0.53–0.82)	0.001	0.66 (0.53–0.82)	0.001
	**DPT 1**			
**Gestation age**				
Late preterm	1		1	
Very preterm	0.65 (0.52–0.81)	0.001	0.68 (0.55–0.87)	0.002
Extremely preterm	0.52 (0.34–0.79)	0.002	0.64 (0.41–1.00)	0.054
**Birth weight**				
Normal	1			
Low birth weight	0.69 (0.28–1.67)	0.406		
Very LBW	0.55 (0.23–1.35)	0.195		
Extremely LBW	0.33 (0.12–0.92)	0.035		
**Maternal education**				
Primary and none	1		1	
Secondary	1.17 (0.89–1.52)	0.24	1.25 (0.95–1.65)	0.108
Tertiary and higher	1.33 (0.94–1.88)	0.112	1.59 (1.11–2.30)	0.012
**Admission to SCBU**				
No	1		1	
Yes	0.09 (0.03–0.26)	0.001	0.11 (0.03–0.32)	0.001
**Length of SCBU stay**	0.98 (0.97–0.99)	0.004	0.98 (0.97–0.99)	0.023
	**DPT 3**			
**Gestation age**				
Late preterm	1		1	
Very preterm	0.68 (0.54–0.86)	0.01	0.66 (0.51–0.85)	0.001
Extremely preterm	0.65 (0.42–0.99)	0.049	0.89 (0.54–1.47)	0.661
**Birth weight**				
Normal	1		1	
Low birth weight	0.70 (0.26–1.90)	0.485	0.64 (0.23–1.76)	0.39
Very LBW	0.54 (0.20–1.45)	0.221	0.53 (0.19–1.45)	0.214
Extremely LBW	0.32 (0.10–0.98)	0.046	0.24 (0.07–0.77)	0.017
**Maternal education**				
Primary and none	1		1	
Secondary	1.17 (0.89–1.53)	0.269	1.29 (0.97–1.71)	0.079
Tertiary and higher	1.53 (1.07–2.18)	0.021	1.99 (1.35–2.94)	0.001
**Length of SCBU stay**	0.98 (0.96–0.99)	0.001		
	**Measles vaccine**			
**Parity**				
<3	1		1	
≥3	0.83 (0.73–0.95)	0.006	0.53 (0.35–0.79)	0.002

### Qualitative results

#### Timing of vaccination among preterm infants

All FGD participants and key informants believed that preterm infants should be vaccinated like any other child, arguing that their immunisation should be prioritised given their increased susceptibility to infections due to prematurity.

“*…We need to immunise premature babies because they are born before they are due*, *they easily get diseases that may be severe compared to term babies due to their weak bodies*.*” (FGD Older caregivers)*

However, primary caregivers’ knowledge about the timing of vaccination of preterm infants was inaccurate. Most of them did not know when preterm infants should receive the various vaccines.

#### Reasons for delaying vaccination among preterm infants

Three main organizing themes related to the preterm infant, health system and parental factors emerged from the discussions as major reasons for delaying vaccination as indicated in (Table 6).

**Table 6:** Reasons for untimely vaccination of preterm infants aged 6–24 months at Mulago Hospital, Kampala, Uganda.

Organizing themes	Sub-themes
Preterm infant factors	• Small size and low weight of the preterm infant • Illness of the preterm infant • Longer stay in the special care baby unit (SCBU) • Being delivered at home or clinic
Health system factors	• Inadequate knowledge of the health worker • Negative perception and attitude of the health workers towards preterm infants • Inadequate communication by the health workers • Stock-out of vaccines • Gaps in documentation of vaccination status e.g. Non issuance of immunisation cards at discharge from the SCBU • Work overload and high number of preterm infants
Parental factors	• Inadequate knowledge of caregivers about vaccination of preterm infants • Fear of side effects

#### Preterm infant factors

**Size and weight of the preterm infant**

The small size and low weight of preterm infants were mentioned as major factors responsible for delaying vaccination. Some caregivers thought that preterm infants were too young to be vaccinated while others feared that the vaccines were not safe especially for very small babies.

“*I had to wait until the baby had gained some weight and looked better before I could take him for immunisation (FGD-Young caregivers)*

Some FGD participants noted that fear to vaccinate preterm infants owing to being small were also prevalent among health workers resulting in delaying vaccination.

“*Although the doctor had told me to immunise the baby*, *the nurse advised me to delay saying*, *“…*. *the baby is only 1kg and you want to immunise*!*” (FGD-Older caregivers)*

#### Sickness of the preterm infant

Sickness of a preterm infant even with minor symptoms like fever and common cold led to postponing or delaying vaccination. It was noted that both caregivers and some health workers especially at peripheral health facilities deferred vaccination of preterm infants when sick.

“*I was told (by health worker) not to take the baby for immunisation when sick*. *I took the baby for immunisation only when she had fully recovered*.*” (FGD-Young caregivers)*

#### Length of stay in the special care baby unit

Prolonged stay of the preterm infant in the SCBU also led to a delay in receiving subsequent vaccines. At the time of the study, the unit only provided the vaccines given at birth, BCG and OPV 0. Irrespective of the length of stay in the SCBU, preterm infants receive subsequent vaccines from the child health clinic of Mulago hospital or other health facility near home after discharge from the SCBU.

“*I spent two months on the ward (SCBU)*, *the baby got the first immunisation but other immunisations were not given*. *At discharge*, *I was told the baby had missed some vaccines and was advised to go to a health facility near home to get them*.*” (FGD-Young caregivers)*

#### Place of delivery

Additionally, not delivering from hospital was another factor that contributed to missing or delaying vaccination among preterm infants.

“*Mothers who deliver at home or in clinics which do not have vaccines miss out on BCG and Polio 0*.*” (KI-Nurse)*

#### Health system factors

**Inadequate health workers’ knowledge**

Inadequate knowledge of health workers regarding care of preterm infants including their vaccination was mentioned as a major factor for their delayed vaccination. Most study participants reported that health workers especially at peripheral health centres rarely encounter preterm infants hence, not sure of when to immunise them.

“*At discharge we ask them (caregivers) to continue immunisation from nearby facilities*, *unfortunately when they bring back the babies to us*, *the information we get is*, *“the health worker refused to immunise*, *the baby is a preterm*.*”(KI-Paediatrician)*

#### Negative attitude of health workers

Some caregivers reported feeling discouraged about returning for subsequent immunisation due to the way they are treated by health workers. Some health workers were said to be abusive towards caregivers and their children and had preferential treatment for term babies. Some felt their babies were not even considered to be children as one caregiver narrated:

“*A woman with a preterm at the health facility was asked by the nurse*, *what is this (referring to the preterm infant) that you have brought here*? *Take it away*! *I advised her to take the baby to another hospital*.*” (FGD-Older caregivers)*

#### Stock out of vaccines

Study participants highlighted stock-out of vaccines as a major hindrance to timely vaccination of their babies. Many narrated how they made multiple visits to health centres to have their babies vaccinated.

“*I went to the health facility multiple times without success*, *I got the polio vaccine on the third attempt and only few people received the vaccine on that day*, *the rest missed*.*” (FGD-Older caregivers)*

#### Inadequate communication by health workers

Some caregivers mentioned that they were not told if their children had been vaccinated while in SCBU. They were not sure of their children’s vaccination status and when to take them for further vaccination after discharge from hospital.

“*When I left hospital*, *I noticed a small mark on the baby’s arm; I thought a mosquito had bitten the baby*. *I was later told the baby had been immunised*.*”(FGD-Young caregivers)*

Key informants confirmed that it was a common practice for preterm infants to be vaccinated while at the SCBU without the knowledge of their caregivers.

“*Whether a baby is immunised or not is written on their discharge letter sometimes*. *But mothers may not know that their child was immunised even if it was written on the discharge letter because they were not informed*.*” (KI-Paediatric resident)*

Inadequate or lack of information given to caregivers about their preterm infants’ vaccination kept them unsure of when to commence or continue immunisation thus leading to delay. It was also noted that sometimes health workers forget to ask about the immunisation status of the preterm infant during subsequent follow up visits mainly because of the busy clinics run by few health workers.

#### Gaps in documentation of vaccination status of preterm infants

Qualitative findings further revealed that vaccinated preterm infants are not issued with immunisation cards at discharge from SCBU. Information regarding vaccines administered while in the nursery is recorded on the discharge letter and the caregiver is encouraged to get the card at the next immunisation. Some parents may remain unaware of the infant’s vaccination status and not know when to vaccinate.

“*At discharge*, *the doctor did not tell me that the baby had been immunised until I asked*. *He told me the baby had received the first immunisation without giving details*. *When I went to the health facility near home*, *I was asked whether the baby had been immunised*, *I told them the baby had received the first immunisation but they did not understand*. *I had no immunisation card from the special care unit*.*” (FGD-Older caregivers)*

#### Parental factors

**Fear of side effects**

Caregivers commonly mentioned fear of side effects as a reason for not vaccinating their preterm infants on time.

“*The baby was a preterm weighing less than 1 kg*, *but they were telling me to immunise the baby*. *The baby had been on oxygen for two months*! *My fears were worse because this was my first baby*. *I had been told that when you immunise a baby they get fever and it can be undesirable*, *I was scared*.*” (FGD-Young caregivers)*

#### Primary caregivers’ knowledge

Some mothers were not knowledgeable about when vaccines should be given and some chose not to vaccinate.

## Discussion

Our study observed that more than half (57%) of preterm infants aged 6–24 months attending a follow up clinic at Mulago National Referral hospital in Uganda did not receive at least one vaccine with in the recommended time ranges. The high proportion of preterm infants with untimely vaccination is worrying given the increasing number of preterm deliveries in Uganda [3, 5] and the fact that these infants are highly susceptible to vaccine preventable diseases. Estimates of timely vaccination in our study are likely to be higher than at lower level health facilities given that the study was conducted at a tertiary hospital where infants are routinely followed up. Untimely vaccination has far reaching implications for both preterm infants and other children in the community. Owing to delayed vaccination, preterm infants are at increased risk of acquiring vaccine preventable diseases with resultant mortality. In addition, children with incomplete vaccinations predispose to outbreaks of vaccine preventable diseases in communities.

Untimely receipt of vaccines affecting various vaccine antigens in our study has also been observed in other studies especially in high-income countries [13, 16, 18].

BCG was the timeliest vaccine with 92% of infants receiving it on time. This estimate is similar to what was found among children in a community-based study in Kampala (92.7%), although these were not preterm infants [34].

Delivery at hospital was significantly associated with timely BCG and OPV 0 receipt in our study, a finding similar to what has been reported in other African settings [34, 36, 37]. In Uganda, administration of BCG and OPV 0 at birth is a requirement for all registered maternity health facilities, which could explain the timely receipt of these vaccines compared to the subsequent ones in the schedule [37].

A discrepancy in the timeliness of BCG and OPV 0 vaccines that are both administered at birth was noted in this study, a finding that was also observed in Kenya although this was not among preterm infants [37]. This disparity could be explained by the polio vaccine stock-outs that were reported by study participants and the EPI representative of the hospital. Vaccine stock outs hamper timely receipt of vaccines among children. In this case, polio vaccine stock-outs have the potential to undermine efforts targeted towards eliminating polio in Uganda. Therefore, the Ministry of Health (MOH) should ensure an adequate supply of all routine vaccines in all health facilities across the country.

The majority of preterm infants had a significant delay starting the first dose of DPT 1, PCV 1 and OPV 1 at 6 weeks compared to the second and third doses at 10 and 14 weeks. Similar findings have been reported among preterm infants in Italy and Netherlands [16, 18], highlighting the fact that this is could be a common trend and problem worldwide. Delayed administration of the first dose usually means that subsequent doses may also be delayed [18]. Therefore, it is extremely important that the first vaccination is given on time. Delaying vaccination increases the period preterm infants are at risk of contracting diseases, which could undermine efforts towards preventing invasive pneumococcal disease, eliminating polio and could result in re-emergency of other VPDs like pertussis in Uganda. In addition, the number of preterm infants missing vaccines increased by the number of doses for vaccines administered at 6, 10 and 14 weeks. This is consistent with findings of delayed uptake of vaccination with increasing age observed in other studies [38,39]. This shows that infants missing vaccines are not completing the recommended vaccine schedule, which in the long run could decrease the country’s vaccine coverage. Therefore, it is crucial that vaccination among preterm infants is started and completed on time.

Untimely DPT 1 and DPT 3 receipt in our study was significantly associated with gestation age, birth weight, length of stay in the SCBU and maternal education level. With the exception of birth weight, these findings are contrary to findings from an Italian cohort of very preterm infants. Tozzi et al found delayed start of DPT 1 was associated with hospitalisation after discharge from the neonatal intensive care unit, high parity and paternal unemployment, factors that were statistically insignificant in our study [16]. Untimely receipt of DPT 1 in our study could be explained by the fact that the SCBU only provides vaccines given at birth (BCG and OPV 0) and not the subsequent vaccinations. We observed that with increasing length of stay in the SCBU, infants were less likely to receive DPT 1 on time. Indeed, mothers with infants who stayed long in the nursery cited this as a reason for late vaccinations of their infants. Our finding is consistent with findings from Switzerland where prolonged hospitalisation was associated with delayed DPT 1 vaccination of preterm infants weighing < 1500g [40]. Extremely LBW (<1,000g) infants had untimely DPT 3 receipt, however, this was significant for DPT 1 at bivariate analysis only. Extremely LBW infants are likely to remain hospitalised for longer after birth due to underlying medical conditions resulting in vaccination delay especially if unstable. Although, vaccination is initiated during hospitalisation of preterm infant in the SCBU of Mulago hospital, provision of subsequent vaccines for infants who stay longer is recommended to minimise delays.

Very preterm infants were vaccinated with significant delay for both DPT 1 and DPT 3. Similar findings have been found in other studies [13, 16, 18, 32] despite a difference in vaccination schedules between countries. This highlights a strong correlation between prematurity and delayed vaccination as previously documented [13, 18]. On the other hand, extreme prematurity was not significant for both DPT 1 and DPT 3 in our study. This is contrary to findings from other studies [13, 18, 32], which could be attributed to the small number of extremely preterm infants in our study.

Low level of maternal education was associated with untimely vaccination of preterm infants in our study for both DPT 1 and DPT 3. This is consistent with findings from other studies that show that having a post-primary education is associated with better utilisation of health care services including immunisation [41–44]. Evidence shows that the levels of knowledge and use of vaccination services are greater for women with at least some secondary education [42]. Although the government of Uganda is providing universal primary education, further investments and political will are required to ensure that majority of children attain at least secondary education in order to improve utilisation of all primary health care services including immunisation.

Only 125/350 (35.7%) preterm infants were 9 months and older and eligible for measles vaccine. Overall, the majority 112/125 (89.6%) of these infants had received measles vaccine on time. Untimely receipt of measles vaccine was associated with increasing parity in our study. Similar findings have been documented in other studies and attributed to the higher cost and demand on resources caused by having many children thus adversely affecting utilisation of healthcare services [34, 37]. With increasing number of siblings, domestic and family responsibilities for the mother or caregiver are likely to increase, with limited attention to the infant, which may result in forgetting the vaccination appointment [44, 45]. Indeed, previous studies have cited “mothers’ being very busy” as a reason for incomplete vaccination of their infants [44–47]. Although, these studies were not conducted among preterm infants, nonetheless, the same explanation may still be plausible. On the other hand, 6 infants received measles vaccine early. Early vaccination has health, cost, administrative and programmatic implications [34]. For example, a study in Netherlands found that while early measles vaccination provides immediate protection, there were concerns of long-term protection [48]. This resulted from an observation that children, who received the vaccine at less than 9 months of age, had their antibodies drop to levels below the cut off required for clinical protection at 4 years [48]. This reduced protection in early-vaccinated children may increase the risk of future potential disease outbreaks [48] and may necessitate providing booster measles doses. The current Ugandan EPI schedule provides for measles vaccination once at 9 months, therefore, additional doses will have cost and programmatic implications. As such, timely vaccination is paramount to avert such consequences and ensure the success of the EPI program in the country.

Qualitative findings further revealed that gaps in documenting vaccination status as evidenced by non-issuance of immunisation cards at discharge from SCBU contributed to untimely vaccination. This finding is in line with those from a previous Ugandan study which revealed that lack of an immunisation card decreased the likelihood of a child being fully immunised [41]. Having an immunisation plan in the form of an immunisation card with a clearly labelled schedule ensures that mothers can easily follow the immunisation schedule. This minimises forgetfulness and enables timely immunisation of their children [41]. Therefore, it is important that all preterm infants are issued with vaccination cards documenting the vaccination status at discharge from hospital.

Some caregivers were often not told if their children had been vaccinated, where and when to get subsequent vaccines, leading to untimely vaccination. Communication plays an important role in disseminating information about important health issues and available health care services [49]. Therefore, effective communication and information transfer between the health workers and the parents is important in ensuring that they follow the immunisation schedule to enable timely vaccination.

Negative attitude of health workers towards caregivers and their children was cited as another reason for untimely vaccination of the preterm infants. Indeed, other studies have documented the role of attitudes and behaviour of the health workers towards mothers as important in determining use of immunisation services [22,49–51]. Unpleasantness of health workers has been highlighted as a major health system barrier influencing vaccination in low middle-income countries [22]. The fear of side effects following immunisation and misinformation especially about immunising a sick child were cited as reasons for untimely vaccination of preterm infants in our study. Concerns about harmful events following immunisation is a major contributor to vaccine hesitancy in both high and low-income countries [19, 22, 23, 25, 26, 49, 51]. As such, it is a key barrier to timely vaccination, reflecting a need to strengthen provision of accurate information to improve vaccine timeliness.

### Study strengths and limitations

Our study was conducted using mixed methods, adding robustness to our findings. Discussions with the parents or guardians and interviews with health workers provided an in-depth understanding of the factors contributing to delayed vaccination of preterm infants at Mulago hospital. To our knowledge, this is the first study to determine the timeliness of vaccination and associated factors among preterm infants in Uganda.

While the quantitative study did not assess vaccination hesitancy, the qualitative study shed light on it as one of the major barriers to timely vaccination of preterm infants. Other factors such as availability of transport, distance to the nearest health facility, maternal illness and maternal immunisation during antenatal care that could influence the timeliness of vaccination of preterm infants were also not assessed. Where vaccination dates of some antigens were not recorded on the child health card or hospital discharge letter, this was documented as ‘not given’ even when the caregiver reported that it was given. Thus the actual timeliness could have been under estimated in our study. However, such cases were very few, thus the underestimation could have been minimal. There is a potential for recall bias in this study, as parents could not remember some information accurately. This was a hospital-based study, as such; the views of caregivers of preterm infants with limited interface with the health care system might be under represented. However, the mixed methods nature of our study generated a wealth of information regarding barriers to timely vaccination of preterm infant, which is important in developing strategies to strengthen vaccination of this highly vulnerable group of infants.

## Conclusion

The majority of preterm infants at Mulago hospital did not receive at least one vaccine on time, despite routine follow up in a specialised clinic by doctors. Untimely vaccination increases susceptibility to vaccine preventable diseases among preterm infants. We recommend that multiple actions should be integrated to improve implementation of existing recommendations. These include, prioritising vaccination of high-risk populations including preterm infants by the government. Issuing vaccination cards indicating vaccination status of the preterm infant at discharge from hospital. Health workers should promote vaccination of preterm infants by regularly checking for their vaccination status during routine follow up visits. Other measures to improve vaccination timeliness among preterm infants in the country include, education of health workers and public sensitisation with clear messages about vaccination of preterm infants, and maintaining an adequate supply of all routine vaccines at all health facilities. Further research is needed to understand the gaps in knowledge, attitudes and practices of health workers regarding vaccination of preterm infants in Uganda.

## Supporting information

S1 Table Bivariate analysis of factors associated with untimely vaccination of preterm infants aged 6–24 months at Mulago Hospital, Kampala, Uganda. (DOCX)
