# Supplementary material for: Vaccination timeliness and associated factors among preterm infants at a tertiary hospital in Uganda
Source: PLoS One. 2019 Sep 6;14(9):e0221902. doi: 10.1371/journal.pone.0221902 (PMC6730875; doi:10.1371/journal.pone.0221902)
Supplement: S1 Table — (DOCX) [file pone.0221902.s001.docx]

**S1 Table. Bivariate analysis of factors associated with untimely vaccination of preterm infants aged 6-24 months at Mulago Hospital, Kampala, Uganda**

| **Variable** | **BCG & OPV0** |  | **DPT1** |  | **DPT3** |  | **Measles** |  |
| --- | --- | --- | --- | --- | --- | --- | --- | --- |
|  | **HR (95% CI)** | **P-value** | **HR (95% CI)** | **P-value** | **HR (95% CI)** | **P-value** | **HR (95% CI)** | **P-value** |
| **Gestation age** |  |  |  |  |  |  |  |  |
| Late preterm | 1 |  | 1 |  | 1 |  | 1 |  |
| Very preterm | 1.08 (0.87-1.35) | 0.475 | 0.65 (0.52-0.81) | 0.001 | 0.68 (0.54-0.86) | 0.01 | 0.71 (0.48-1.05) | 0.083 |
| Extreme preterm | 1.09 (0.70-1.60) | 0.791 | 0.52 (0.34-0.79) | 0.002 | 0.65 (0.42-0.99) | 0.049 | 1.09 (0.54-2.22) | 0.811 |
| **Birth weight** |  |  |  |  |  |  |  |  |
| Normal | 1 |  | 1 |  | 1 |  | 1 |  |
| Low birth weight | 0.98 (0.40-2.38) | 0.959 | 0.69 (0.28-1.67) | 0.406 | 0.70 (0.26-1.90) | 0.485 | 0.60 (0.15-2.49) | 0.485 |
| Very LBW | 0.94 (0.39-2.30) | 0.899 | 0.55 (0.23-1.35) | 0.195 | 0.54 (0.20-1.45) | 0.221 | 0.57 (0.14-2.39) | 0.443 |
| Extremely LBW | 0.54 (0.19-1.54) | 0.247 | 0.33 (0.12-0.92) | 0.035 | 0.32 (0.10-0.98) | 0.046 | 0.67 (0.14-3.19) | 0.616 |
| **Gender** |  |  |  |  |  |  |  |  |
| Male | 1 |  | 1 |  | 1 |  | 1 |  |
| Female | 1.08 (0.87-1.33) | 0.477 | 1.03 (0.84-1.28) | 0.754 | 1.14 (0.91-1.42) | 0.258 | 1.18 (0.82-1.69) | 0.371 |
| **Delivery place** |  |  |  |  |  |  |  |  |
| Hospital | 1 |  | 1 |  |  |  | 1 |  |
| Home or clinic | 0.72 (0.52-0.99) | 0.047 | 0.71 (0.52-0.98) | 0.04 |  |  | 1.19 (0.64-2.22) | 0.586 |
| **Multiple births** |  |  |  |  |  |  |  |  |
| No | 1 |  | 1 |  |  |  | 1 |  |
| Yes | 1.10 (0.87-1.40) | 0.436 | 1.33 (1.05-1.69) | 0.017 |  |  | 1.03 (0.68-1.55) | 0.892 |
| **Admission to SCBU** |  |  |  |  |  |  |  |  |
| No | 1 |  | 1 |  |  |  |  |  |
| Yes | 0.51 (0.18-1.38) | 0.185 | 0.09 (0.03-0.26) | 0.001 |  |  |  |  |
| **Length of SCBU stay** | 0.99 (0.98-1.00) | 0.206 | 0.98 (0.97-0.99) | 0.004 | 0.98 (0.96-0.99) | 0.001 | 1.01(0.99-1.03) | 0.224 |
| **Re-admission to SCBU** |  |  |  |  |  |  |  |  |
| No | 1 |  | 1 |  |  |  | 1 |  |
| Yes | 0.98 (0.75-1.28) | 0.88 | 0.92 (0.71-1.21) | 0.561 |  |  | 1.29 (0.83-2.00) | 0.254 |
| **Maternal age** | 1.00 (0.99-1.02) | 0.661 | 1.01 (0.99-1.02) | 0.588 | 1.01 (0.99-1.03) | 0.183 | 0.99 (0.96-1.03) | 0.589 |
| **Maternal education** |  |  |  |  |  |  |  |  |
| Primary | 1 |  | 1 |  | 1 |  | 1 |  |
| Secondary | 0.99 (0.77-1.29) | 0.967 | 1.17 (0.89-1.52) | 0.24 | 1.17 (0.89-1.53) | 0.269 | 1.06 (0.67-1.66) | 0.81 |
| Tertiary and higher | 1.23 (0.87-1.74) | 0.239 | 1.33 (0.94-1.88) | 0.112 | 1.53 (1.07-2.18) | 0.021 | 1.49 (0.82-2.69) | 0.192 |
| **Parity** | 0.98 (0.92-1.05) | 0.68 | 0.98 (0.92-1.06) | 0.719 |  |  | 0.83 (0.73-0.95) | 0.006 |
| **Maternal employment** |  |  |  |  |  |  |  |  |
| No | 1 |  | 1 |  |  |  | 1 |  |
| Yes | 1.07 (0.86-1.32) | 0.543 | 1.08 (0.88-1.35) | 0.439 |  |  | 1.19 (0.82-1.72) | 0.356 |
| **ANC attendance** |  |  |  |  |  |  |  |  |
| No | 1 |  | 1 |  |  |  |  |  |
| Yes | 0.84 (0.43-1.64) | 0.612 | 0.58 (0.29-1.12) | 0.105 |  |  |  |  |
| **Vaccine stock out** |  |  |  |  |  |  |  |  |
| No | 1 |  | 1 |  |  |  | 1 |  |
| Yes | 0.66 (0.53-0.82) | 0.001 | 1.02 (0.82-1.27) | 0.847 |  |  | 0.93 (0.64-1.34) | 0.684 |
